# Students’ Perceptions of Instructional Rubrics in Neurological Physical Therapy and Their Effects on Students’ Engagement and Course Satisfaction

**DOI:** 10.3390/ijerph18094957

**Published:** 2021-05-06

**Authors:** Rafael García-Ros, Maria-Arantzazu Ruescas-Nicolau, Natalia Cezón-Serrano, Juan J. Carrasco, Sofía Pérez-Alenda, Clara Sastre-Arbona, Constanza San Martín-Valenzuela, Cristina Flor-Rufino, Maria Luz Sánchez-Sánchez

**Affiliations:** 1 Department of Developmental and Educational Psychology, Faculty of Psychology, University of Valencia, 46010 Valencia, Spain; Rafael.Garcia@uv.es; 2 Physiotherapy in Motion, Multispeciality Research Group (PTinMOTION), Department of Physiotherapy, University of Valencia, 46010 Valencia, Spain; Natalia.Cezon@uv.es (N.C.-S.); Juan.J.Carrasco@uv.es (J.J.C.); Sofia.Perez-Alenda@uv.es (S.P.-A.); clarasastre90@hotmail.com (C.S.-A.); M.Luz.Sanchez@uv.es (M.L.S.-S.); 3 Intelligent Data Analysis Laboratory, ETSE (Engineering School), University of Valencia, 46100 Burjassot, Spain; 4 Unit of Personal Autonomy, Dependency and Mental Disorder Assessment, Faculty of Medicine, University of Valencia, 46010 Valencia, Spain; Constanza.Martin@uv.es; 5 Research Unit in Clinical Biomechanics–UBIC, Department of Physiotherapy, University of Valencia, 46010 Valencia, Spain; 6 Centro Investigación Biomédica en Red de Salud Mental, CIBERSAM, 28029 Madrid, Spain; 7 Department of Physiotherapy, University of Valencia, 46010 Valencia, Spain; Cristina.Flor@uv.es

**Keywords:** rubrics, physical therapy, formative assessment, validity, usefulness, students’ engagement, course satisfaction, students’ perceptions

## Abstract

One of the main challenges faced by physical therapy (PT) students is to learn the practical skills involved in neurological physical therapy (PT). To help them to acquire these skills, a set of rubrics were designed for formative purposes. This paper presents the process followed in the creation of these rubrics and their application in the classroom, noting that students perceived them as valid, reliable, and highly useful for learning. The perception of the validity and usefulness of the rubrics has different closely related dimensions, showing homogeneous values across the students´ sociodemographic and educational variables, with the exception of dedication to studying, which showed a significant relationship with schoolwork engagement and course satisfaction. The adequacy of the hypothesized structural model of the relationships among the variables was confirmed. Direct effects of the perception of the rubrics’ validity and engagement on course satisfaction were found, as well as direct effects of the assessment of the usefulness of the rubrics on schoolwork engagement and indirect effects on course satisfaction through this latter variable. The results are discussed taking into account the conclusions of previous research and different instructional implications.

## 1. Introduction

In the field of physical therapy (PT), as in other healthcare disciplines, professionals have to master competencies from different specialties [1]. One of these specialties, neurorehabilitation, is particularly difficult, given the breadth, diversity, and complexity of the problems it addresses. Neurological conditions present diverse symptoms and a prolonged and variable time course, and they can cause complex disabilities, including physical, cognitive, behavioral, and communication deficits [2]. In addition, rehabilitation from neurological diseases is based on neuroplasticity [3], or the nervous system’s ability to functionally and physically change or restructure in response to environmental stimuli, cognitive demands, or behavioral experiences [4]. Thus, understanding adaptive behavior in response to nervous system injury requires knowledge about the interaction between the body and the environment, as well as the feedback loop involving the nervous system, the body, and the environment [2]. 

In addition, many studies highlight the importance of developing manual skills in a broad set of PT subjects because they are essential in the professional world [5,6,7]. In the PT degree, they are usually studied in laboratory classes taught by different professors [8]. This is the case of neurological PT, where students have to acquire and fluidly apply a wide range of different techniques and maneuvers [9,10,11], devote ample time to their practice, and apply them repeatedly and with different participants to achieve sufficient variability in their practice [12]. In addition, the process of learning neurorehabilitation techniques and maneuvers is more demanding than in other PT areas, given its greater breadth, diversity, and specificity, an issue highlighted by the students [13]. Consequently, it is particularly relevant to provide students with different types of support to promote their learning. In this regard, instructional or formative rubrics can be particularly useful resources because they provide students with the criteria and performance levels to be reached. Rubrics also allow teachers to carry out frequent formative assessments and provide higher quality feedback, and they promote self-regulated learning [14]. Moreover, different studies show that students value rubrics as guidelines for their autonomous work [15]. This paper focuses on these aspects, presenting the development process and application of a set of formative rubrics designed to provide support in learning the various neurological PT maneuvers taught in the PT degree. The main objective of the study was to evaluate these rubrics from the students’ perspective by determining their assessment of the rubrics’ validity and usefulness, as well as the effects of the rubrics on students’ engagement and course satisfaction.

### 1.1. Assessment Rubrics in University Studies

A rubric is an assessment tool that can be defined as “a coherent set of criteria for students’ work that includes descriptions of performance levels for the criteria” [16]. Recently, the use of rubrics in university education has increased considerably, both from the perspective of summative assessment as a grading tool and from the perspective of enhancing formative assessment by guiding students in learning and developing skills [17,18,19,20,21,22,23], which is the perspective adopted in this study. 

More specifically, reviews of research on assessment rubrics [17,18,19,20] highlight that the studies can be classified into three main groups depending on their objectives: 

(a) Studies carried out from the perspective of using rubrics in summative assessment. They focus on determining the quality of the information provided by rubrics for evaluating/grading by analyzing their reliability [21,24,25,26] and/or validity [21,27,28], concluding that rubrics make it possible to increase the validity, consistency, and reliability of grading.

(b) Studies carried out from the perspective of formative assessment that view rubrics as instructional or teaching tools. They focus on the effects of rubrics on students’ learning outcomes [19,29] and/or levels of self-regulated learning and motivation [19,30,31,32,33]. Among their conclusions, it is worth mentioning that rubrics increase the transparency of the assessment process, improve the quality of the feedback provided by the teachers, and enable students to perform more accurate self- and peer-assessments, thus helping to achieve better learning outcomes. 

(c) The third group, in which the present study is framed, analyzes students’ and teachers’ experiences, perceptions, and attitudes related to the quality, use, and usefulness of rubrics [18,20]. These studies are particularly relevant, given that students’ perceptions and attitudes influence the way rubrics are used in the classroom [34,35,36]. In addition, in most cases, rubrics are created by the teachers, and so it is necessary to find out whether the students understand, value, and use them [19,20,34]. These studies conclude that university students use rubrics and find them useful, especially formative rubrics, and they view them as more than just grading tools [15,18,19,33,35,37,38,39,40]. However, they also indicate that merely providing students with rubrics does not guarantee that they will use them or obtain any learning benefits [19]. Instead, it is necessary to consider several essential aspects when creating rubrics and using them in the classroom—e.g., involving students in their development, demonstrating their understanding and positive assessment as learning guides—[18,34,36,37,41,42]. Fewer studies analyze teachers’ perceptions and attitudes about rubrics, and they conclude that teachers mainly view them as more objective grading tools, but with limited formative value [18,20,43].

### 1.2. Research on Rubrics in PT Studies

Studies analyzing the usefulness of rubrics in PT studies are scarce, compared to other healthcare areas (e.g., medicine, nursing, psychology) [44,45]. In these disciplines, numerous studies have evaluated their usefulness for assessing and developing research skills [46,47], critical thinking and clinical case analysis skills [28], and/or technical and clinical case management competencies [48,49]. Their findings concur with those previously highlighted [44,50], emphasizing the development of complex skills and the integration of theoretical and practical training, especially in the area of clinical competencies [22,34,44]. 

Focusing specifically on PT, several studies highlight the relevance of having valid and reliable instruments to assess clinical competencies in different training contexts [51,52]. Thus, recent studies show adequate interrater reliability in the application of a rubric designed to assess undergraduate students’ use of different therapies for musculoskeletal disorders [53], the moderate internal validity of a rubric—Case History Assessment Tool (CHAT)—to assess clinical reasoning in graduates [54], or the adequate reliability and validity of a rubric—Measurement Tool for Clinical Competencies in PT (MTCCP)—designed to evaluate clinical competencies in a professional context [55]. 

Other studies have analyzed the validity and usefulness of various rubrics that assess the information literacy skills of graduate and postgraduate health sciences students, including PT students. Turbow and Evener [56] found that a modified version of the information literacy Valid Assessment of Learning in Undergraduate Education (VALUE) is appropriate for assessing the information literacy skills of graduate health sciences students, although they also highlight its low interrater reliability in grading clinical case reports. Turbow et al. [57] reach similar conclusions about the usefulness of an adaptation of the VALUE written communication rubric. In a subsequent review paper, Boruff and Harrison (2018) [58] point out that librarians are often involved in the development and evaluation of information literacy skills in PT training courses. They emphasize the need for valid and reliable rubrics that can add greater rigor to their evaluations. These authors highlight that many of the available rubrics are too simple and not very useful for evaluating clinical case reports written by students, or they are too complex because they require very specialized knowledge [59]. Thus, they conclude that Turbow and Evener’s proposal [56] is the most suitable for librarians, although it is necessary to specify the criteria for assessing different types of tasks (e.g., critical evaluation of topics, research projects, clinical case reports, etc.) in greater detail.

According to Furze et al. [60], a rubric to assess the clinical reasoning skills of undergraduate PT students makes it possible to test the level and rate of acquisition of these skills, providing faculty with information about the effectiveness of their instructional strategies. Gamel et al. [61] analyze the reliability and usefulness of a systematic literature review rubric (SLR-Rubric) for graduate students, confirming its suitability and receiving positive evaluations from students and professors. Chong et al. [22] analyze the usefulness and student ratings of a set of rubrics for learning clinical skills related to prescribing and teaching therapeutic exercises to patients. The students emphasize the importance and usefulness of the rubrics as support in ongoing formative assessment. In addition, the analysis of access to rubrics and their use shows that they promote self-regulated learning, foster students’ self-assessment of their progress and online feedback, and significantly and positively correlate with academic results. Martiañez et al. [62] analyze undergraduate students’ perceptions of the usefulness of three rubrics (clinical histories, clinical cases, and reflexive diaries) that evaluate the competencies of the clinical PT internship. They conclude that students view rubrics as moderately useful, that providing rubrics to students does not guarantee that they will perceive them as valid and use them to learn, and that rubrics should be considered basic referents throughout the teaching-learning process.

In the field of neurological PT, Del Rossi et al. [63] analyze the usefulness of a rubric—Interprofessional Collaborator Assessment Rubric (ICAR)—designed to evaluate interprofessional skills involved in pediatric collaborative practices. Their results highlight the rubric’s usefulness in assessing these skills and helping students to identify the quality criteria involved in authentic learning activities that are similar to real-life practices. Finally, Tappan et al. [64] describe the process of creating a set of rubrics to assess four different vestibular rehabilitation skills on the practical exam for an entry-level PT doctoral program, showing satisfactory levels of interrater agreement in their use.

### 1.3. Process of Development and Use of Neurological PT Rubrics

Studies that analyze students’ experiences, perceptions, and attitudes towards rubrics give special importance to the description of the development process and the use of rubrics in the classroom [18]. The three professors of the neurological PT course in the PT degree program at the University of Valencia participated in the study. They all have extensive clinical and training experience in this field. A researcher with expertise in educational psychology also participated in the research group. In the development process, four additional PT faculty members and seven students provided input about the rubric’s clarity and comprehensibility. The principles highlighted in the research on formative rubrics and good use practices were followed during their creation and subsequent use [19,32,65,66]. The following phases were followed in developing the rubric: 

(a) Initial analysis and decision-making. Prior to beginning to create the rubrics, the teachers agreed: (a) through consensus, to develop a set of rubrics to be used in the formative and summative assessment of neurological PT maneuvers (neurodevelopment, proprioceptive neuromuscular facilitation—PNF—and infant PT), thus promoting their content validity and alignment with the objectives and competencies of the subject matter; (b) to develop analytical rubrics, making it possible to provide students with feedback about the different specific criteria considered in their performance; (c) to develop rubrics that integrate similar criteria for the different types of maneuvers, in order to foster their recall; (d) to develop four proficiency levels for the criteria (from inadequate to advanced); (e) along with the rubrics, to incorporate verbal guidelines for the steps to follow in each maneuver, in order to encourage self-assessment and peer assessment, provide more specific feedback, and promote subsequent review by the students. Based on their greater specialization in neurological PT, two professors jointly developed an initial draft of the rubrics on neurodevelopment (18 maneuvers) and infant PT (7 maneuvers), whereas a third professor developed the initial version of the rubric on PNF (9 maneuvers).

(b) Determining the criteria, performance levels, and grading strategy. Based on the initial drafts, the criteria to be considered in the rubrics were discussed and agreed upon. After several discussion cycles, the following criteria were finally considered: position of the physical therapist, position of the patient, verbal guidance in performing the maneuver, fluidity, and execution of the maneuver.

(c) After determining the criteria, each professor was asked to assign a relative weight to each criterion in the grading strategy, agreeing that all of them would have the same value in the total score when evaluating the execution of the maneuvers. Each professor individually elaborated an initial description of the proficiency levels (from inadequate to advanced) of the performance criteria, agreeing that their attributes should be specified in terms of the intensity and adequacy of their application (e.g., performs all the holds adequately). Finally, and depending on the teacher’s specialization, responsibilities were assigned for developing the verbal guidelines for the maneuvers. The final wording of the proficiency levels and guidelines was also determined by consensus.

(d) Assessment by professors of other courses and students. The initial versions of the rubrics were presented to a group of four professors from other specialties and seven students in the degree program. They had to rate the rubrics’ usefulness for formative and summative assessment in the course, the comprehension and adequacy of the criteria, and the performance levels, terminology, and grading system to be used. The results were satisfactory, and minor modifications were made.

(e) Consistency in the application of the rubrics. Prior to their use in the classroom, in order to unify the application criteria, the teachers separately assessed the execution of nine different maneuvers that had been video-taped by a group of students from the previous course. The discussion of the individual assessments made it possible to increase the correspondence between them (an example of the original definitive version of a rubric is available in Table S1).

(f) Explanation and modelling of their use in the classroom. The rubrics and guidelines for performing the maneuvers were provided and explained to the students on the first day of the practical classes, modeling and exemplifying their use for practicing and learning the maneuvers. Their importance and usefulness for formative assessment and learning was emphasized, as well as their use as grading tools in the course (summative assessment). 

(g) Use of the rubrics. In the successive practical sessions in the course, the rubrics were used for the analysis, assessment, and discussion of the level of the students’ performance on the maneuvers (working in pairs, alternating the role of physical therapist and patient). They were also used systematically by the faculty to model the maneuvers and provide feedback to the students. Students also used them throughout the course to self-regulate their learning, self-assess their progress, and carry out peer-assessments. Finally, to improve their instructional use, small adjustments were made in their wording based on feedback from the students after using them.

(h) Final assessment, revision, and improvements. The rubrics were used in the final assessment of the subject to record the errors made on the different criteria for eight different maneuvers. At the end of the academic year, work meetings were held to analyze the results obtained and the students’ ratings of the validity and usefulness of the rubrics and suggest possible improvements in their content and use.

### 1.4. Objectives and Hypotheses

The rubrics were incorporated into the neurological PT course as learning support (formative assessment) and grading tools (summative assessment), considering the following study objectives:

(a) Determining the students’ ratings of the rubrics created, in terms of their validity and reliability as assessment tools and their usefulness for learning, and identify potential areas for improvement in the rubrics (criteria, performance levels, and grading strategy) and their instructional use.

(b) Determining the relationship between students’ ratings of the assessment rubrics and their levels of schoolwork engagement and course satisfaction in the neurological PT course. 

Based on these objectives, the study hypotheses are the following:

(a) Students will rate the rubrics positively, in terms of facilitating the learning of the maneuvers (formative assessment) and validly and reliably assessing their performance levels (summative assessment), given that the research principles for good practices in the development and use of rubrics in the classroom were followed [34,66].

(b) A significant relationship will be found between the students’ ratings of the rubrics and their levels of engagement [67,68,69] and course satisfaction [70], and between these latter two variables [71]. The relationship between student’ engagement and academic satisfaction has been repeatedly confirmed in previous research based on the most prevalent theoretical models of student engagement [72,73]: models that consider students’ cognitive, behavioral, and emotional engagement dimensions [74,75]; models that add a fourth agentic dimension [76,77]; and the prevailing student engagement model in Europe, which considers the vigor, dedication, and absorption dimensions [78,79,80]. 

**Figure 1:**
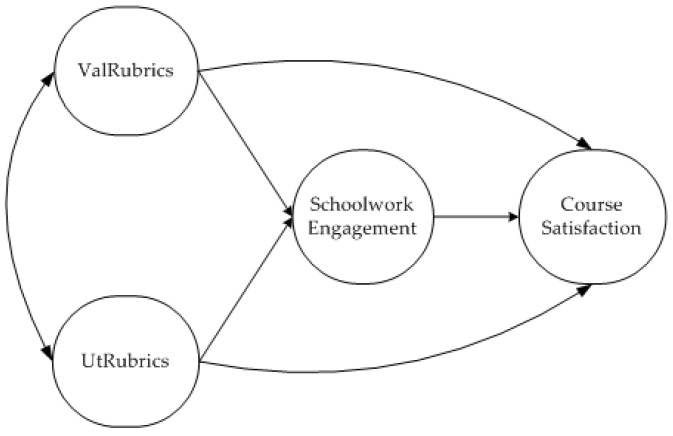
Hypothesized structural model. ValRubrics: rubrics’ validity; UtRubrics: rubrics’ usefulness.

Figure 1 shows the hypothesized structural relationships among the study variables. In the structural model, the following hypotheses stand out: (1) students’ perceptions of the rubrics’ validity and usefulness will have a significant effect on their schoolwork engagement; (2) students’ engagement will have a significant effect on course satisfaction; (3) students’ schoolwork engagement will partially mediate the effect of students’ perceptions of the rubrics on their course satisfaction.

## 2. Materials and Methods

### 2.1. Study Design and Procedure

A cross-sectional survey study was carried out. The Ethics Committee of the University of Valencia approved the research protocol for the study (Code H1543332503311). The study inclusion criterion was to be a PT student in Clinical Specialties IV course, which addresses the Neurorehabilitation contents, in the third year of the PT Degree at the University of Valencia in the 2017-18 academic year. The class lasts one semester, and its practical part consists of 21 face-to-face hours that take place in laboratories in groups of 16–18 students.

In the first week of May 2018, an email was sent to students inviting them to respond to an anonymous online survey about their perceptions of the validity and usefulness of the rubrics used in the class, as well as their levels of engagement and satisfaction with the course. Questions related to students’ sociodemographic and educational variables were also included, as well as an open-ended question related to aspects of the course and rubrics that could be improved. The first page of the survey described the study characteristics and objectives and requested students’ informed consent to complete the survey.

Of the 173 students enrolled in the course, 127 responded to the questionnaire (response rate of 73.41%). Their mean age was 21.96 years (SD = 3.30; range = 19–38 years), with a similar distribution of sex (55 females and 72 males). Of the total number of participants, 80.31% were full-time students, and 81.1% had entered university studies through the baccalaureate degree and EBAU tests.

### 2.2. Measures

Perception of validity and usefulness of the assessment rubrics (*PVURE*). Considering the principles and guidelines for the construction and use of rubrics in the classroom [18,19,81], a questionnaire was created to rate the perception of the validity and reliability of the rubrics (7 items), as well as their usefulness and use in learning the maneuvers (10 items). A five-point Likert-type response scale was used (1 = “Strongly Disagree”; 5 = “Strongly Agree”).

Schoolwork Engagement Inventory (SEI-EDA). Schoolwork engagement was assessed with the SEI-EDA [79,80], derived from the UWES-9 scale [78]. The SEI-EDA has nine items that measure Energy (e.g., “At university, I am bursting with energy”), Dedication (e.g., “I am enthusiastic about my studies”), and Absorption (e.g., “Time flies when I am studying”), with regard to schoolwork. The SEI-EDA also makes it possible to obtain a global score for schoolwork engagement that is used in this study. The responses are rated on a five-point scale ranging from 1 (never) to 5 (always). In previous studies, the scale showed adequate internal consistency (α = 0.83). In this study, the scale also showed satisfactory psychometric characteristics (α = 0.87, CRI = 0.84, EVA = 0.58, ω = 0.87).

Course Satisfaction. This was evaluated with the satisfaction with the university context subscale of the Multidimensional Students’ Life Satisfaction Scale (MSLSS) [82,83]. This subscale includes eight items that evaluate university students’ satisfaction with the academic environment. In this study, the term “university” was substituted with “in this course” (e.g., "I like the activities we do in this course"). The subscale has a five-point Likert-type response scale (1= “Strongly disagree”; 5 = “Strongly agree”). Its internal consistency in previous studies was 0.80 [82,84], and it also showed satisfactory reliability levels in this study (α = 0.87, CRI = 0.86, EVA = 0.60, ω = 0.87). 

Finally, a questionnaire developed ad hoc for the study was administered to collect the participants’ sociodemographic and educational variables (dedication to study, university entrance modality and grade, GPA in the degree, and courses pending from previous years). The questionnaires were followed by an open question about aspects of the course and the rubrics that could be improved.

### 2.3. Analysis

As a previous analysis, the factorial structure of the *PVURE* was determined through confirmatory factor analysis techniques (CFA), applying the Robust Maximum Likelihood method with the EQS 6.1 program [85]. The objective was to determine whether students’ ratings of the validity/reliability and usefulness of the rubrics could be considered a single dimension or two related dimensions. To analyze this question, two alternative structural models were tested using the Satorra–Bentler Chi-square statistic [86], the comparative fit index (CFI) [87], the non-normalized fit index (NNFI) [87], and the root mean square error of approximation (RMSEA) [88], with its 90% confidence interval. CFI and NNFI values equal to or greater than 0.90 indicate adequate fit levels [89]. RMSEA values below 0.05 indicate a good fit, and values in the 0.05–0.08 range indicate a reasonable fit. The reliability of the resulting dimensions was determined through their internal consistency (Cronbach’s alpha).

To determine the students’ perception of the validity and usefulness of the rubrics, the basic descriptions of the items and the dimensions underlying the *PVURE* (*ValRub* and *UtRub*) were obtained. Subsequently, we analyzed the relationship and possible significant differences in the ratings of the rubrics based on the students’ sociodemographic and educational variables by means of Pearson’s correlation coefficient and different *t*-tests. 

Finally, using a structural equations methodology, the hypothesized structural model relating students’ assessment of the rubrics to schoolwork engagement and satisfaction with the course was tested. For this purpose, item parcels were established on the scales used in the study, considering 2 or 3 adjacent items: three parcels in *ValRub* (average of items 1-2, 3-4, and 5-6-7), five in *UtRub* (average of items 1-2, 3-4, 5-6, 7-8, and 9-10), and four in both SEI (items 1-2, 3-4, 5-6, and 7-8-9) and MSLSS (items 1-2, 3-4, 5-6, 7-8). Establishing item parcels produces more stable solutions, better fit levels, fewer biases, and smaller estimation errors [90].

## 3. Results

### 3.1. Previous Analyses. Factorial Structure of the PVURE

To determine the structure of the *PVURE*, two alternative structural models were considered: a one-dimensional model (M1) whose items assessed a single underlying factor; a two-factor oblique model (M2), with the first factor related to the perceived validity and reliability of the rubrics (*ValRub*)—integrating the items focusing on this issue (items 1–7)—and the second factor related to the use and usefulness of the rubrics (*UtRub*) for learning (items 8–17). 

The results reveal that M1 does not show an adequate fit to the data (SB χ^2^(119) = 259.5, *p* < 0.01; NNFI = 0.813; CFI = 0.790; RMSEA = 0.097, 90% CI (0.080–112)), whereas M2 provides a satisfactory representation of the participants’ responses (SB χ^2^(117) = 160.9, *p* < 0.01; NNFI = 0.924; CFI = 0.934; RMSEA = 0.055, 90% CI (0.031–0.074)). All the items show high factorial saturations in their corresponding dimensions (range 0.59–0.90), and the two dimensions show a high correlation with each other (r = 0.81, *p* < 0.001) and satisfactory psychometric characteristics (*ValRub*: α = 0.92, CRI = 0.93, AVE = 0.64, ω = 0.92; *UtRub*: α = 0.94, CRI = 0.94, AVE = 0.65, ω = 0.94). In short, the *PVURE* makes it possible to obtain the students’ assessment of the validity and reliability of the rubrics (*ValRub*) and their usefulness for learning (*UtRub*), with the two dimensions demonstrating adequate psychometric characteristics and a close relationship with each other.

### 3.2. Rating of the Validity/Reliability and Usefulness of the Rubrics

Table 1 shows that all the *ValRub* items present means close to or greater than four. The overall mean for *ValRub* is 4.12. Higher scores were obtained on the items “integrates the most important elements to consider in the maneuvers” (M = 4.36; SD = 0.85), “helps to understand the criteria involved in proper execution” (M = 4.26; SD = 0.85), and “makes it possible to evaluate the important competencies in this area” (M = 4.20; SD = 0.88). In contrast, lower scores were found for “integrates criteria that will be useful to me in my professional future.” (M = 3.76; SD = 1.11).

Table 2 highlights the results for *UtRub*, showing that the overall mean score on the usefulness of the rubrics is 4.13. The items with the highest ratings are “to better know the criteria they were going to use to assess us” (M = 4.49; SD = 0.78) and “to guide the study/practice of the maneuvers” (M = 4.28; SD = 0.93). The item "to reduce my anxiety in the process of learning the maneuvers" (M = 3.35; SD = 1.28) presents the lowest score. 

The analysis of the relationships between *ValRub* and *UtRub* and the students’ sociodemographic and educational variables showed the absence of significant differences in their evaluations based on gender (*ValRub*: t(125) = −1.26, *p* = 0.21: *UtRub*: t(125) = −1.27, *p* = 0.21), and the absence of significant relationships with their age, university access modality and entrance grade, and academic results in their studies. Significant differences were obtained in *ValRub*, but not in *UtRub*, based on dedication to studying (*ValRub*: t(125) = −2.97, *p* < 0.01: *UtRub*: t(125) = −1.42, *p* = 0.15), with full-time students giving higher ratings.

### 3.3. Relationships between the Perception of the Rubrics and the Educational Outcomes

Finally, the hypothesized structural model of the relationships and effects of students’ ratings of the rubrics (*ValRub* and *UtRub*) on schoolwork engagement and course satisfaction was evaluated. The relationships between the dimensions were all significant in the hypothesized direction. Thus, higher ratings of the rubrics were related to greater engagement (*ValRub*, r = 0.47, *p* < 0.001; *UtRub*, r = 0.52, *p* < 0.001) and course satisfaction (*ValRub*, r = 0.51, *p* < 0.001; *UtRub*, r = 0.46, *p* < 0.001), which were also significantly related to each other (r = 0.72, *p* < 0.001).

**Figure 2:**
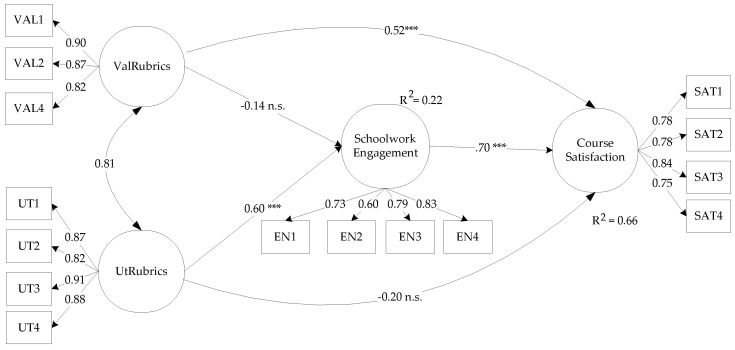
Standardized parameter estimates in the model.Item parceling was used for the latent variables of rubrics’ validity (ValRubrics), rubrics’ usefulness (UtRubrics), Schoolwork engagement, and Course satisfaction, with parcels made with adjacent items; all parameter estimates were statistically significant (*** *p* < 0.001), unless otherwise (ns = non-significant) stated.

The hypothesized structural model satisfactorily represents the data (SB χ^2^_95_ = 102.71, *p* = 0.27; RMSEA = 0.025, 90% CI (0.000–0.055); CFI = 0.990; NNFI = 0.988). Parameter estimates for the model are shown in Figure 2. Significant direct effects of *UtRub* on schoolwork engagement are observed, as well as of *ValRub* and schoolwork on course satisfaction. In addition, significant indirect effects of *ValRub* on course satisfaction through schoolwork engagement are observed (β = 0.41, *p* < 0.001). In other words, according to the working hypotheses, schoolwork engagement partially mediates the effects of the students’ perception of the rubrics on their course satisfaction. Lastly, Figure 2 shows that the model explains 22% of the variance in schoolwork engagement and 66% of the variance in course satisfaction.

### 3.4. Difficulties in Learning the Maneuvers and Improvements in the Rubrics

The analysis of the responses to the open question on the questionnaire showed that the students’ main difficulties in learning the maneuvers were: (a) the breadth and variety of maneuvers to be learned (highlighted by 40 students), (b) the difficulty of executing them (23 students, especially in relation to performing correct holds and PNF maneuvers), (c) the level of specificity and detail involved in their performance (21 students), and (d) the need for more time and opportunities to practice (14 students). Regarding improvements in the rubrics, the students mentioned the need to include visual aids/videos to help them learn the maneuvers (18 students).

Finally, the students’ performance levels on eight maneuvers from the final test of the course material were recorded. The performance levels shown were adequate (M = 11.66; SD = 1.7; grading scale between 0–15 points), although there was little variability in the grades for the Fluidity criterion on all the maneuvers (in 92% of the cases, the highest proficiency level was given). In addition, the most common errors were related to the criteria associated with executing the maneuvers (specifically the holds) and, to a lesser degree, the verbal guidance during the maneuvers.

## 4. Discussion

The main objectives of this study were (1) to analyze the students’ ratings of the validity and usefulness of a set of rubrics designed to help them learn the maneuvers involved in neurological PT and more objectively rate the performance level of these techniques, and (2) to evaluate the relationship between these ratings and two especially relevant educational outcomes in psychoeducational research, schoolwork engagement and course satisfaction. These two outcomes are closely linked to learning outcomes and academic performance in university studies, perseverance until earning the degree, and students’ psychological well-being [91,92,93].

The initial analyses found that the perceptions of the validity and reliability of the assessment rubrics (*ValRub*) and their usefulness for learning (*UtRub*) are different but closely related dimensions. Thus, a greater perception of the validity and reliability of the rubrics is directly related to a higher assessment of their usefulness for promoting learning. These results are congruent with previous research [18,20]. If rubrics are viewed as integrating and reliably assessing important competencies in academic subjects or in the professional field using clear and appropriate criteria, they will also be valued as useful learning tools that support formative and summative assessment. In contrast, if students think rubrics are more related to the teacher’s demands than to the criteria for the tasks, they will consider them of little use for learning [33] or more focused on grades than on learning [20,94].

In relation to the first hypothesis, the results showed that the students rate the rubrics as valid, valuable, and practical tools for learning the neurorehabilitation maneuvers. Thus, they highly rate almost all the indicators related to the rubrics’ validity and reliability in assessing the quality and performance levels for implementing the maneuvers, as well as their usefulness for achieving better learning outcomes. These results are congruent with previous research, given that in the development and application of the rubrics, the principles and recommendations for good practices in the elaboration and use of rubrics in the classroom were followed [19,32,34,64,65,66]. Thus, for example, they were developed with the consensus of all the teachers of the subject, who had extensive clinical experience and training in neurological PT (content validity). Moreover, their use in the classroom was explained and modelled, verifying that students understood the criteria and quality levels to be considered in their application. Furthermore, they were used as a basic reference in the feedback given by the faculty, and students were encouraged to perform frequent self-assessments to check their progress.

Regarding the perceived validity and reliability of the rubrics, the results were satisfactory. Particularly noteworthy were the ratings for “integrates the most important elements to consider in the maneuvers”, “helps to understand the criteria involved in proper performance”, “allows the assessment of important competencies in this area”, or “is a reliable tool (makes it possible to measure the quality of the maneuvers)”. The lowest mean rating, although adequate, was obtained by the indicator "makes it possible to evaluate important skills for my professional future”. This last question is easily understood, given that neurorehabilitation is only one of the specialties in the students’ PT degree, and they tend to find it more complicated and demanding than other professional areas and courses required in the degree [13]. In sum, students’ ratings show that the rubrics have high content validity and allow them to reliably assess their performance levels on the maneuvers.

The results for the rubrics’ usefulness for promoting and guiding learning are also very satisfactory. Particularly noteworthy are the ratings of their usefulness for “better knowing the criteria they are going to use to assess us”, “guiding the study/practice of the maneuvers”, “clarifying how we had to perform each maneuver”, and “being able to perform the maneuvers with greater quality”. These results also coincide with previous research indicating that rubrics increase the transparency of the assessment process, serve as a guide for developing learning tasks, and foster self-regulation and better results [18,19,20]. In contrast, the indicator with the lowest rating is “decreases my anxiety in the process of learning the maneuvers”, which is also often highlighted as a positive effect of the formative use of rubrics [19,33]. With regard to this question, three complementary aspects can be pointed out. First, the value of this indicator is significantly higher than the mean value of the response scale used, and so it can be considered adequate, although it is certainly lower than the ratings of the other indicators. Second, previous research indicates that students perceive that neurological PT is a particularly complex and difficult subject, and so the level of anxiety when it is assessed can be higher than in the other degree subjects [13]. Finally, the large number of maneuvers to be learned in this subject, as well as their difficulty and specificity, requires a considerable amount of practice that can also be related to greater anxiety before the final evaluation. In any case, this indicator is an aspect that can be improved by increasing the opportunities to learn the maneuvers in the classroom and during the students’ autonomous work time, making improvements in the instructional methodology and increasing the diversity of the learning activities (e.g., group discussions on applying the maneuvers when performed by students and recorded on video), and/or creating new web-based instructional resources (e.g., video modelling the maneuvers) [19].

In addition, the perception of the rubrics as valid, valuable, and practical tools extends to all the students, with no significant differences depending on their sociodemographic and educational characteristics. Significant differences were only obtained for *ValRub*, with results quite similar to *UtRub*, depending on the dedication to studying (full-time vs. part-time), with full-time students rating the rubrics more positively. This result makes sense because full-time students can practice the maneuvers more frequently and regularly and perform more self-assessments and peer-assessments, even though part-time students also view them as valid and useful tools for learning. These results coincide with previous research, although more research is needed [19], given that several studies indicate that their use may affect the self-efficacy levels of men and women differently [33] or that women state that rubrics have a greater impact on their learning levels [15].

In relation to the second study hypothesis, and congruent with previous research, the results show that students who rate the rubrics as more valuable and practical for promoting learning also demonstrate greater schoolwork engagement [67,69,95,96] and course satisfaction [70]. In turn, these last two educational outcomes are significantly related to each other, as found in numerous studies conducted with university students and at other educational levels [71,72,91,92].

More specifically, the hypothesized structural model predicted that the perceived validity and usefulness of the rubrics would show significant direct effects on students’ academic engagement and course satisfaction, as well as effects of engagement on course satisfaction. In addition, it proposed the existence of significant indirect effects of rubric assessments on course satisfaction through schoolwork engagement. The results highlight the model’s capacity to satisfactorily explain the students’ responses.

Thus, first, the results showed significant positive direct effects of schoolwork engagement, considered a key indicator of the quality of university education and defined as the amount of time and effort students put into their studies and other educationally purposeful activities [92], on course satisfaction. These results are similar to what has been found in various previous studies with university students [91,97,98,99,100,101]. Second, the findings showed that the perceived usefulness of rubrics has direct positive effects on schoolwork engagement and indirect effects on course satisfaction through the latter, whereas the perceived validity and reliability of rubrics has direct effects on course satisfaction. These results are consistent with previous research, given that (a) employing active instructional practices in the classroom and fostering formative assessment (e.g., promoting self-regulated learning, providing guidelines for performance, emphasizing self-assessment and facilitating awareness of the progress made, and providing students with more detailed and personalized feedback) are significant predictors of both academic engagement and course satisfaction [69,102,103]; and (b) the instructional methodology and assessment rubrics employed, both for summative and formative purposes (e.g., clarity, representativeness, and alignment between the learning objectives and their criteria, specifying performance standards to be achieved, fostering fair and equitable assessment of students) are significant predictors of satisfaction with university courses in a wide variety of disciplines [68,70,95,104,105]. In summary, consistent with the study hypotheses, we found significant positive direct effects of *ValRub* and schoolwork engagement on course satisfaction, and of *UtRub* on schoolwork engagement, which mediates the effects of *UtRub* on course satisfaction.

Finally, also congruent with the previous research, students highlight the difficulty of learning the breadth, diversity, and complexity of the maneuvers involved in neurological PT. They propose integrating visual/video supports with the rubrics in order to facilitate their learning. Moreover, the results found in the final course evaluation suggest the need to make modifications in the criteria related to the fluidity and performance of the maneuvers. From this same perspective, as previously highlighted, once the appropriate modifications have been made in the rubrics, it will also be relevant to analyze and improve the evidence of their reliability (e.g., internal and inter-rater reliability) and validity (e.g., criterion and construct validity), in order to increase the quality of the information they provide for summative assessment or grading purposes.

The study limitations include the sample size and the fact that it was carried out in a single university, thus limiting the generalizability of the conclusions. It would be interesting to analyze how students perceive the use of assessment rubrics in larger samples of neurological PT students and in both undergraduate and graduate courses. In addition, assessment of the perception of the validity and usefulness of the rubrics at different times during the academic year would have allowed us to check the progression in the assessment of the rubrics, as well as possible variations in their relationship with schoolwork engagement and course satisfaction over time. It would also have been especially interesting to analyze their predictive capacity of academic performance, an issue that was not considered in this study. An additional limitation is that course satisfaction, as in most of the studies with university students that analyze this variable [91,106,107,108], has been considered an educational outcome in the university, but it could also be considered a determinant of the level of student engagement. Finally, it would also be interesting to analyze the ratings and effects of rubrics provided through different media and/or information presentation channels (e.g., physical vs. electronic, visual vs. verbal) on students’ schoolwork engagement, satisfaction, and learning outcomes.

## 5. Conclusions

The results show that the students positively rate the validity and usefulness of a set of rubrics created to aid in learning neurological PT maneuvers, considering the principles and good practices highlighted in previous research in their development and use [25,34].

In agreement with the study hypotheses, a significant positive relationship was found between the students’ ratings of the rubrics and their schoolwork engagement and course satisfaction. The adequacy of the hypothesized structural model of the relationships between the study variables was also demonstrated, highlighting the significant direct effects of the perception of the validity and reliability of the rubrics and schoolwork engagement on course satisfaction, as well as the significant direct effect of the perception of the usefulness of the rubrics on schoolwork engagement and its indirect effect on course satisfaction through schoolwork engagement.

These conclusions coincide with previous studies that emphasized the importance of analyzing students’ perceptions and attitudes about the quality, validity, and usefulness of assessment rubrics [18] because their attitudes determine how rubrics are used and to what degree. In addition, the conclusions highlight the usefulness of formative rubrics for learning complex skills, as well as the need to consider the principles and good practices pointed out in previous research in their development and application [19,34].

## Figures and Tables

**Table 1:** Descriptive statistics for *ValRub*.

I Think the Rubric …	Mean	SD	Min	Max	Sk	Ku
1. Integrates the most important elements to consider in the maneuvers	4.32	0.85	1	5	−1.7	3.5
2. Makes it possible to evaluate the important competencies in this subject	4.20	0.88	1	5	−1.3	2.1
3. Integrates criteria that will be useful to me in my future professional career	3.76	1.11	1	5	−0.8	0.1
4. Is a reliable tool (makes it possible to measure the quality of the execution)	4.12	0.94	1	5	−1.2	1.6
5. Clearly highlights and differentiates the levels considered in each criterion	4.07	0.90	1	5	−1.2	1.9
6. Fosters a fair comparison of the different students on the practical assessment test	4.09	0.99	1	5	−1.1	0.9
7. Helps to understand the criteria involved in adequate performance	4.26	0.85	1	5	−1.4	2.7
Total	4.12	0.78	1	5	−1.5	3.2

ValRub: rubrics’ validity; SD = Standard Deviation; Min = Minimum; Max = Maximum; Sk = Skewness; Ku = Kurtosis.

**Table 2:** Descriptive statistics for *UtRub.*

I Think the Rubric is Useful for	Mean	SD	Min	Max	Sk	Ku
1. Clarifying how we have to perform each maneuver	4.22	0.90	1	5	−1.1	0.9
2. Planning the study/practice of the maneuvers	4.14	0.92	1	5	−0.8	0.1
3. Reviewing what is learned in order to make adjustments	4.17	0.90	1	5	−0.9	0.5
4. Realistically rating the execution of the maneuvers	4.17	0.92	1	5	−1.2	1.7
5. Guiding the study/practice of the maneuvers	4.28	0.93	1	5	−1.4	2.1
6. Discussing and determining what to improve in their execution	4.08	0.95	1	5	−1.1	1.1
7. Being able to perform the maneuvers with greater quality	4.21	0.94	1	5	−1.1	1.0
8. Facilitating the study/practice of the maneuvers	4.21	0.89	1	5	−1.2	1.4
9. Knowing more about the criteria that will be used to assess us	4.49	0.78	1	5	−1.7	3.1
10. Reducing my anxiety in the process of learning the maneuvers	3.35	1.28	1	5	−0.3	−0.9
Total	4.13	0.77	1	5	−1.2	1.8

UtRub: rubrics’ usefulness; SD = Standard Deviation; Min = Minimum; Max = Maximum; Sk = Skewness; Ku = Kurtosis.

## Data Availability

The data underlying this article will be shared on reasonable request to the corresponding author.

## Supplementary Materials

The following are available online at https://www.mdpi.com/article/10.3390/ijerph18094957/s1, Table S1. Example of the definitive version of an original rubric.
