# Supplementary material for: Students’ Perceptions of Instructional Rubrics in Neurological Physical Therapy and Their Effects on Students’ Engagement and Course Satisfaction
Source: Int J Environ Res Public Health. 2021 May 6;18(9):4957. doi: 10.3390/ijerph18094957 (PMC8125510; doi:10.3390/ijerph18094957)
Supplement: Supplementary file 1 [file ijerph-18-04957-s001.zip › ijerph-1197094-supplementary.pdf]

Table S1. Example of the definitive version of an original rubric

**Maneuver: Change from sitting postural set to supine postural set**									
From the correct sitting postural set (see rubric no.6), the PT raises the stretcher to the level of the pat´s greater trochanter and stands on the pat's paretic side.	**The PT facilitates movement from the pat's CKP, by placing the caudal hand at the front and the cranial hand behind.**	**The PT creates a forward rotational movement of the CKP by bending the pat´s trunk while facilitating its rotation towards the less affected side.**	With the caudal hand (previously placed on the CKP), the PT descends the pat´s trunk towards the stretcher.	**With the elbow of the cranial arm, the PT keeps the pat's head in slight flexion throughout the descent movement to the stretcher (avoiding its hyperextension).**	At the last moment, before the pat reaches the stretcher, the PT brings the pat's trunk towards him- or herself and places it parallel to the stretcher Doing so, the PT facilitates lifting the pat´s less affected LL to the stretcher to finish this movement.	The PT places the pat's head gently on the pillows, avoiding hitting it in extension.	**The PT grabs the pat´s foot in neutral dorsiflexion with the caudal hand, and stabilizes the most affected knee with the cranial hand, while asks the pat to raise the paretic LL onto the stretcher.**	The pat is placed in the appropriate supine postural set using the bridge maneuver.	The technique is performed fluently

**RUBRIC:**				
	**INADEQUATE (1)**	**IMPROVABLE (2)**	**ADEQUATE (3)**	**ADVANCED (4)**
**Physical therapist position**	The student is not placed on the correct side of the stretcher and the height of the stretcher is also incorrect.	The student is not placed on the proper side of the stretcher but the height of the stretcher is correct.	The student stands on the correct side of the stretcher, but the height of the stretcher is not correct.	The student stands on the correct side of the stretcher and the height of the stretcher is correct.
**Position of the subject with hemiparesis**	At the beginning, the subject sitting postural set is incorrect and at the end, the subject supine postural set is also incorrect.	The subject starting and ending postural sets are practically correct (except for one or two components).	The subject starting and ending postural sets are correct but the student does not explain the rationale for them.	The subject starting and ending postural sets are correct and the student fully explains the rationale for them.
**Verbal facilitation of the maneuver**	The subject voluntary activity is not facilitated because the maneuver or the activity to be carried out is not indicated.	The subject voluntary activity is scarcely facilitated since only some activity to be carried out is indicated.	Most of the subject voluntary activity is facilitated but the student forgets to indicate the execution of one or two activities.	The subject voluntary activity is totally facilitated, always indicating the maneuver to be carried out and the activity to be performed.
**Fluency**	The student does not identify the maneuver to be performed and gets stuck without knowing what to do.	The student manages to identify the maneuver to be performed after taking one minute to remember it or performs it intermittently, as has to stop to think every step over.	The student takes a few seconds to remember the maneuver but performs it smoothly and continuously without hesitation.	The student identifies the maneuver immediately and performs it smoothly and continuously without hesitation.
**Execution of the maneuver**	The student performs the maneuver sequence improperly and his/her holds are not correct (grasp the paretic ankle or wrist, instead of holding the paretic foot and hand in a functional position).	The student performs the maneuver sequence properly but gripping is not correct (grasps the paretic ankle or wrist, instead of holding the paretic foot and hand in a functional position).	The student performs the maneuver sequence properly with the correct gripping, though there are some handling mistakes during the execution.	The student handles the subject perfectly and performs the maneuver sequence properly with perfect gripping, which rationale is well Known.

PT: physical therapist, pat: patient, LL: lower limb, CKP: central key point, UL: upper limb.
